# Cultural landscapes of the Araucaria Forests in the northern plateau of Santa Catarina, Brazil

**DOI:** 10.1186/s13002-015-0039-x

**Published:** 2015-06-09

**Authors:** Anna Jacinta Machado Mello, Nivaldo Peroni

**Affiliations:** Post-Graduate Program in Ecology (PPGECO) and Human Ecology and Ethnobotany Laboratory – Federal University of Santa Catarina, Center of Biological Sciences, Department of Ecology and Zoology, University Campus João David Ferreira Lima, Córrego Grande, Florianópolis, CEP 88040-900 Santa Catarina Brasil; Federal University of Santa Catarina, Center of Biological Sciences, Department of Ecology and Zoology, University Campus João David Ferreira Lima, Córrego Grande, Florianópolis, CEP. 88040-900 Santa Catarina Brasil

**Keywords:** *Caívas*, Cultural landscapes, Landscape ethnoecology, Historical ecology, Araucaria forests, *Caívas*, Paisagens culturais, Etnoecologia da paisagem, Ecologia histórica, Florestas com araucária

## Abstract

**Background:**

The Araucaria Forest is associated with the Atlantic Forest domain and is a typical ecosystem of southern Brazil. The expansion of *Araucaria angustifolia* had a human influence in southern Brazil, where historically hunter-gatherer communities used the *pinhão*, araucaria’s seed, as a food source. In the north of the state of Santa Catarina, the Araucaria Forest is a mosaic composed of cultivation and pasture inserted between forest fragments, where *pinhão* and *erva-mate* are gathered; some local communities denominate these forest ecotopes as *caívas*. Therefore, the aim of this study is to understand how human populations transform, manage and conserve landscapes using the case study of *caívas* from the Araucaria Forests of southern Brazil, as well as to evaluate the local ecological knowledge and how these contribute to conservation of the Araucaria Forest.

**Methods:**

This study was conducted in the northern plateau of the state of Santa Catarina, Brazil in local five communities. To assess ethnoecological perceptions the historical use and management of *caívas,* semi-structured interviews, checklist interviews and guided tours were conducted with family units.

**Results:**

In total 28 family units participated in the study that had *caívas* on their properties. During the course of the study two main perceptions of the ecotope *caíva* were found, there is no consensus to the exact definition; perception of *caívas* is considered a gradient. In general *caívas* are considered to have the presence of cattle feeding on native pasture, with denser forest area that is managed, and the presence of specific species. Eleven management practices within *caívas* were found, firewood collection, cattle grazing, trimming of the herbaceous layer, and erva-mate extraction were the most common. *Caívas* are perceived and defined through the management practices and native plant resources. All participants stated that there have been many changes to the management practices within *caívas* and to the *caíva* itself.

**Conclusions:**

These areas still remain today due to cultural tradition, use and management of plant resources. Through this cultural tradition of maintaining *caívas* the vegetation of the Araucaria Forest has been conserved associated to the use of the Araucaria Forests native plant resources.

## Resumo

**Introdução:**

A Floresta Ombrófila Mista, também conhecida como Floresta com Araucária, está associado ao domínio da Mata Atlântica e é um ecossistema típico do sul do Brasil. A expansão da *Araucaria angustifolia* teve uma influência humana no sul do Brasil, onde as comunidades de caçadores-coletores utilizavam o pinhão como fonte de alimento. No norte do estado de Santa Catarina, a Floresta com Araucária é um mosaico, composto por cultivo e áreas de pasto inseridas entre fragmentos florestais, aonde o pinhão e erva-mate são coletados; algumas comunidades locais denominam alguns ecótopos florestais como *caívas*. Portanto, o objetivo desse trabalho é entender como as populações humanas transformam, manejam e conservam as paisagens usando o estudo de caso de *caívas* no sul do Brasil, assim como avaliar o conhecimento ecológico local e como estes contribuem para a conservação da Floresta com Araucária.

**Métodos:**

Este estudo foi realizado no planalto norte do estado de Santa Catarina, Brasil, em cinco comunidades locais. Para acessar a percepção etnoecológica local, e o uso e manejo histórica de caívas, foram feitas entrevistas semi-estruturadas, entrevista *checklist*, e visitas guiadas com unidades familiares.

**Resultados:**

No total, 28 unidades familiares que tem caívas participaram do estudo. Durante o estudo foram encontradas duas principais percepções de caívas, não existe um consenso para a definição, a percepção de caívas é considerada um gradiente. Caívas são considerados como tendo a presença de gado, com área manejada de floresta mais densa, e com a presença de espécies específicas. Foram encontradas onze práticas de manejo dentro de caíva, as comuns foram a recolha de lenha, gado mantidos em pastagens nativas no sub-bosque, o corte do estrato herbáceo, e a extração de erva-mate. Caívas são percebidos e definidos através das práticas de manejo e recursos vegetais nativos da Floresta com Araucária. Todos os informantes afirmaram que houve muitas mudanças nas práticas de manejo dentro de caívas.

**Conclusões:**

Essas áreas permanecem ainda hoje, devido à tradição cultural, uso e manejo dos recursos vegetais. Através desta tradição cultural de manter caívas a vegetação da Floresta com Araucária foi conservado associado ao uso dos recursos vegetais nativos.

## Background

Different vegetation associations classified through the lens of local ecological knowledge are called ecotopes in landscape ethnoecology [1]. Ethnoecologists use the concept of landscape to conduct studies on how humans interpret “local conceptions of landscape”, landscape patterns and classifications, and some study the “local knowledge systems for ecological sustainability” [2, 3]. According to the World Heritage Convention and UNESCO, cultural landscapes are separated into three categories, the first category being a clearly defined landscape designed and created intentionally by humans, the second an organically evolved landscape, and the third an associative cultural landscape [4]. The second category, an organically evolved landscape has within it two subcategories, where cultural landscapes can be defined as relicts or continuing landscapes. Relicts are landscapes whose evolutionary process came to an end, and continuing landscapes are those that continue to evolve and retain an active social role in contemporary society closely associated with the traditional way of life, while exhibiting evidence of evolution over time [4]. The term cultural landscape may also be used to describe how people view, use and occupy their land [2, 5].

Forests around the world in large part have been transformed into cultural landscapes [6], since many forest landscapes are influenced by natural disturbances, as well as by human disturbances [7, 8]. The vegetation patterns, which result from disturbances, reflect complex interactions between biotic and abiotic characteristics [7], as well as cultural characteristics [6]. For example, the Brazilian Amazon is considered more of a garden [9] where biodiversity and landscape features have been transformed through many years of traditional management systems [10, 8]. These traditional management systems and use of biodiversity has transformed many environments into cultural landscapes, shaped overtime by cultural forces that in large part are responsible for the current patterns of biodiversity [9].

Traditional communities generally have large repertoire of ecological knowledge and many communities recognize certain ecogeographic areas or landscapes units [11] based on the principal sets of vegetation, or plant associations [2, 12]. In Mexico, indigenous groups recognize and use landscape units in the environment where they live, within each landscape unit different products can be found; for example, the Huastecos recognize nine landscape units in tropical forests [11]. In the Brazilian Amazon, the Baniwa of the Upper Rio Negro recognize distinct habitats, with specific vegetation in which they classify associations of different biotic characteristics [12]. The Kayapo of the Amazon region use 16 different terms to categorize different vegetation in the amazon forest [13]. Many of these landscape units are described through associations with vegetation, topography, type of soil, ecological indicators, fauna, hydrology, and through different types of use carried out in each area [11].

Forests are not merely viewed as timber resources but also places with non-timber forest products; this can be attributed to the understanding of traditional management practices, and the consistency of human practices with landscape and biodiversity conservation [6]. There are many types of forest management, which can range from specific species management to large-scale management of timber, along with secondary succession management, agroforestry, management of non-timber forest products, as well as others. Cultural forces of ecosystem land use drive many of these management practices.

In the Brazilian Atlantic Forest, there are many local populations, which depend on the extraction and management of natural resources for their survival and livelihood [14, 15]. Local populations not only depend on tropical forests for use of natural resources but also as a source of income [16]. In many regions around the world, these communities and their traditional management systems contribute to local ecosystem and biodiversity maintenance [17, 18].

Within this domain, the Araucaria Forest is one of the associated ecosystems, which is characterized by the presence of the species *Araucaria angustifolia* [19]. This ecosystems’ area has been significantly reduced due to logging, deforestation and expansion of urban areas [20, 21]. The Araucaria Forest covers a major part of the state of Paraná, and extensive areas in the states of Santa Catarina and Rio Grande do Sul [19]. Currently, in southern Brazil, less than 25 % of the original area of Araucaria Forest still exists [22].

In the northern plateau of Santa Catarina the Araucaria Forest landscape is a mosaic formed by forest fragments in between cultivation areas [23]. The Araucaria Forest has been transformed and changed since the end of the Holocene [24, 25]. After the nineteenth century, the native species, *Ilex paraguariensis* (*erva-mate*) became highly valued economically for many human populations, who depended on this resource as a source of income [26, 27]. Along with the extraction of erva-mate began the management of livestock in the understory of the Araucaria, and the exploitation of both species contributed to the formation of a typical system called *faxinal* [28, 29]. The *faxinal* is considered a traditional system that allows the survival of various plant communities and from a landscape perspective is an ecologically viable system [29].

The *faxinal* does not exist as a management system in the state of Santa Catarina; however, *caívas* have similar current and historical management practices. Marques and collaborators [30] describe *caívas* as an “ecosystem made up of native forests - with different densities - whose herbaceous strata is composed of native and/or naturalized pastures that are extensively grazed”. *Caívas* can be seen as landscape units or ecotopes with tree strata of the Araucaria Forest and herbaceous layer composed of pastures, where the livestock are raised and erva-mate is extracted [26].

There is not much information on the floristic composition, structure, and management of *caívas*. There also is no consensual definition for *caívas* within scientific literature, as well as among local communities.

This study sought to answer how human populations conserve and transform forest landscapes through use, and management? We hypothesized that the through the use and management of landscapes the local populations have not only transformed the landscape to promotes species which are used but continue to conserve these forest areas because of the plant resources importance and use within the household. Therefore, the aim is to understand how human populations transform, manage and conserve landscapes using the case study of *caívas* from the Araucaria Forests of southern Brazil. More specifically, we aim to characterize this cultural ecotope through the study of the perceptions of local populations in regards to use, management and used species. Furthermore, this study seeks to exemplify how local populations have conserved these spaces of Araucaria Forests through use and management of landscapes currently and historically.

## Methods

### Study area

This study was conducted in six communities in four municipalities in the northern plateau of the state of Santa Catarina: Campininha, Barra Grande and KM 6 in the municipality of Três Barras; Colônia Escada in the municipality of Irineópolis; Colônia Ruthes in the municipality of Major Vieira; and Forquilhas in the municipality of Canoinhas.

**Fig. 1:**
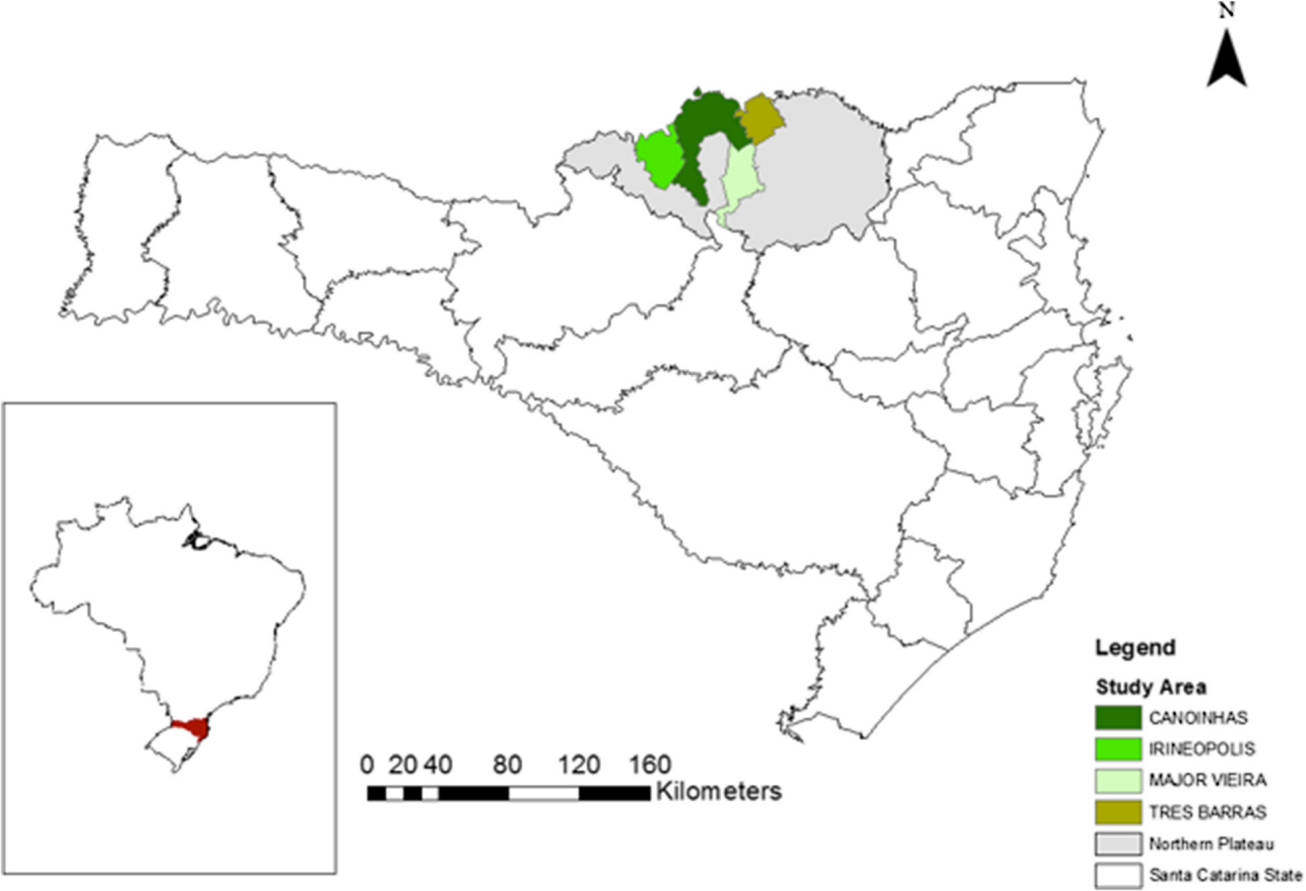
Map of study area in the Northern Plateau of Santa Catarina. Campininha, KM 6 and Barra Grande are located in the municipality of Três Barras, Colônia Escada is located in the municipality of Irinéopolis, Forquilhas is located in the municipality of Canoinhas, and Colônia Ruthes is located in the municipality of Major Vieira. (Constructed with ArcGis by Juan Manuel Otalora & Anna Jacinta Machado Mello)

The communities of Campininha, Barra Grande and KM 6, located in the municipality of Três Barras (Fig. 1) were founded in the 19th Century [31, 26]. The area was mainly used for logging, cattle, and *erva-mate* extraction [26]. There are various immigrant ethnicities in the region, including Polish, a smaller number of Germans, Italians and Lebanese.

The second community, Colônia Escada, is located in the municipality of Irineópolis (Fig. 1). Beginning in 1885 immigrants of various descents, such as German, Polish, Ukrainian, and to a lesser extent Italian, began to colonize the area that is known as Irineópolis. The primary source of income for people of Colônia Escada is agriculture.

Canoinhas and Major Vieira were colonized by *tropeiros* in 1880, who crossed from Rio Grande do Sul to São Paulo transporting cattle and became interested in the rich land and thus established roots in what was then called Colônia Vieira [19]. After the *Contestado* War both areas were colonized primarily by Polish immigrants, and then by immigrants of German, Italian, Ukrainian and Japanese descent during the early 1900’s because of the erva-mate [31].

### Data collection

Within each community participants were accessed based on their willingness to participate and the presence of *caívas* on their properties. The informant sample was increased using the “snow-ball” method [32, 33]. Semi-structured interviews were conducted at the household level and this is considered the sample unit (family unit), on average three people in each family unit were present, ranging from 1 – 8 people. The interview contained both structured questions and open-ended questions regarding the local ecological knowledge, management, and resources from *caívas*.

Before each interview we obtained a prior informed consent in accordance with the code of ethics of the International Society of Ethnobiology and a legal Provisional Measure (MP n° 2.186-16 - 23/08/2001) [34, 35]. The study was also approved by the ethics committee of the Federal University of Santa Catarina (CAAE: 01262212.5.0000.0121).

A “checklist-interview” [36] of 20 priority plants was carried out to access local ecological knowledge on the most used and managed plants. The list of plants was derived from a former project (*Conservabio*^1^ project), and corresponds to the 20 most important non-timber forest resources that are used and managed from the Araucaria Forest according to local communities. These twenty plants are those that were listed as the most used and managed within the community. For a more details on how the priority list was obtained please see the Conservabio Project [see ref. 56 and 57]. For each species all family units were asked to cite current use, historical use, parts of plant utilized, frequency of use, and availability of the resource. The species from the list were: Caraguatá (*Bromelia antiacantha* Bertol.), Espinheira-santa (*Maytenus ilicifolia* Mart. ex Reissek & *Maytenus boaria* Molina.), Araucaria (*Araucaria angustifolia* (Bertol.) Kuntze), Cataia (*Drimys brasiliensis* Miers.), Erva-mate (*Ilex paraguariensis* A.St.-Hil.), Pau-de-andrade (*Persea major* (Meisn.) L.E.Kopp), Bracatinga (*Mimosa scabrella* Benth.), Cedro (*Cedrela fissilis* Vell.), Guavirova (*Campomanesia* sp.), Cambará (*Gochnatia polymorpha* (Less.) Cabr.), Cerninho (*Curitiba prismatica* (D.Legrand) Salywon & Landrum), Cuvatã (*Cupania vernalis* Cambess), Guamirim (*Myrcia* sp.), Imbuia (*Ocotea porosa* (Nees) Barroso), Pau-amargo (*Picramnia parvifolia* Engl.), Pitanga (*Eugenia uniflora* L.), Araça (*Psidium cattleianum* Sabine), Ariticum (*Annona* sp.), Canela guiaca (*Ocotea puberula* (Rich.) Nees), and Aroeira (*Schinus terebinthifolius* Raddi).

After each interview a guided tour was conducted with each informant in order to collect, identify and verify plant material mentioned during the interview [33]. The collection of botanical material followed the standard procedure for ethnobotanical studies [7], and the species were identified through bibliography and consultation with botanical experts using the APGII system (Angiosperm Phylogeny Group II system) of plant classification. Experts at the National Institute of Forestry in São Paulo and the University of São João Del-Rei in Minas Gerais identified Lauraceae and Myrtaceae. Voucher specimens of plants were deposited in the collection of the Human Ecology and Ethnobotany Laboratory at the Federal University of Santa Catarina, and the FLOR Herbarium at the Federal University of Santa Catarina, Brazil. The Brazilian System of Authorization and Information of Biodiversity (SISBIO) authorized the collection of plant material emitted on January 7th 2012 (case number: 32055–1).

### Data analysis

Data analysis consisted of a qualitative description and use of descriptive statistics. The answers were separated into themes, or similar answers, and organized into tables utilizing direct information from the household interviews. The botanical material was used to verify if the plants named in the interview were of the same taxonomic species for all informants.

The answers from the “checklist interview” were organized into a table following Campos & Ehringhaus [36]. For the current and historical use species were sorted into five categories: timber/firewood, medicinal, animal consumption, edible (food & drink), and tools. For the frequency of use each plant was sorted into three categories: always uses (1), sometimes uses (2), almost never uses (3). The availability of the plant was separated into three categories: very abundant (1), not abundant (2) and does not exist (3). The proportion of use, frequency, and availability were calculated for each category following Campos & Ehringhaus [36]. Some participants said they did not use a plant or did not know the plant so they were not included within the calculated proportion. A nine-cell analysis was designed to compare availability of the plant in *caívas* with its current frequency of use.

## Results

In total 28 family units (91 people) participated in this study, three were from the community of Colônia Escada, two from Forquilhas, two from Colônia Ruthes, one from KM 6, eight from Barra Grande, and 12 from Campininha. The average female age in households was 53.3 ranging from 20 to 75 years of age. The average male age was 56, ranging from 26 to 82 years of age. Participants were culturally mixed mostly of Polish, German, Italian and Turkish descent, as well as mixed between African and Indigenous people (*Caboclos*).

The property sizes ranged from 2 ha–50 ha, with an average of 15 ha. Out of 28 family units 26 have properties larger than 1 ha. The average size of *caívas* on these properties was 8.5 ha, ranging from 0.2 ha to 45 ha.

The main source of income for family units is agriculture, and the main crops planted are beans, corn, tobacco, and soybeans. Some of these families also plant potato, wheat, rice and medicinal plants, as well as *Pinus* spp*.* and *Eucalyptus* spp. Families live primarily from retirement benefits, agriculture, as employees of agro and forest companies, maintenance crew of the National Forest of Três Barras, rural tourism, cattle raising for milk, poultry farming, erva-mate extraction, and beekeeping.

### Local perceptions and characterization of the ecotope caívas

When participants were asked if they knew the origin of the word *caíva*, all participants stated that it was a word that had always been used by their parents and grandparents and therefore they continued to use the word. Three family units (11 %) guessed that it might be an indigenous word, but were uncertain. The word *caíva* actually comes from the Tupi language, a now extinct indigenous language, and means “earth improper for cultivation” [37].

**Fig. 2:**
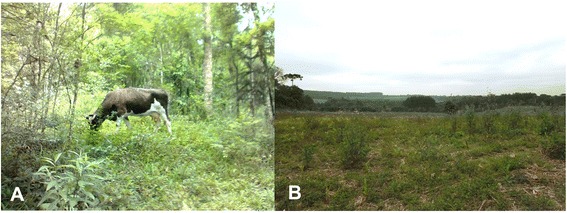
**a**-**b**. Examples of the *caíva* landscape. Demonstration of the two extremes of the perception gradient found in the communities of KM6, Barra Grande, Campininha, Colônia Escada, Forquilhas, and Colônia Ruthes located in the Northern Plateau of the state of Santa Catarina, Brazil. **a** Illustration of the first perception gradient for *caíva*, picture taken in the community of Colônia Escada; **b** llustration of the second perception gradient for *caíva*, picture taken in the community of Barra Grande

The local perception of *caívas* falls into a gradient where two extremes can be defined. Figure 2a, b elucidate these two extreme perceptions of the gradient. The first part of the gradient perception cited by 21 (75 %) family units, is centered around three main resources found within *caívas*: raising cattle on native pastures within forest areas, erva-mate (*Ilex paraguariensis*) extraction, and the presence of araucarias (*Araucaria angustifolia*) and taller but not dense vegetation. The second half of the gradient perception of *caíva* is the exact opposite and was cited by seven family units (25 %). People holding this type of perception considered the first type of perception to refer to general forest cover, where some management is exerted, but considers a *caíva* to be originating from areas of “*roça de toco”* management. An area from “*roça de toco”* is considered a forest area cleaned for swidden cultivation, where the *tocos* (stumps) are left, the area is burned, and traditional agricultural crops are planted among the stumps. After this area is used for cultivation it is left alone for many years so that the natural vegetation may return (fallow). Two family units who were closer in views with this perception said their parents always called them to “clean the *caíva*”.

The family units that fit in closer to the first perception were property owners with large properties, around 15 ha. These families were generally those that had a higher socioeconomic status, in terms of land ownership. These were also families who employed members of the family units holding the second perception. Those who thought *caívas* are fallow areas from swidden cultivation (*roça de toco*), were generally those who were employed in erva-mate extraction, trimming/slashing and caring for the land and cultivation.

There is no consensus to the exact definition of the first perception, therefore perception of *caívas* is considered a gradient. In general *caívas* are considered to have the presence of cattle feeding on native pasture, with denser forest area that is managed, and the presence of specific species, such as *I. paraguariensis* and *A. angustifolia*. There were a few differences where 11 % said the presence of cattle was not necessarily found in a *caívas*, whereas 64 % said that what makes a *caíva* is the presence of cattle. About 61 % of family units stated that a *caíva* is a forest area where the herbaceous layer was removed/mowed in order to for cattle to graze on the native pastures. However, all 21 (75 %) family units stated that without use and management the area is no longer considered a *caíva*.

There was also no consensus within second perception gradient. In general most family units holding a definition closer to the second perception of *caívas* said it was a place where swidden cultivation was performed, and dense vegetation was cleared to plant crops, after which the area was leftover for the native vegetation to grow again. One informant said the difference between forest area and a *caíva* was the presence of specific species, such as bracatinga (*Mimosa scabrella* Benth.). Another informant stated that a caíva is “*terra de plantar*” or cultivation area, with very short non-dense vegetation that could also have the presence of araucária (*Araucaria angustifolia*). Within the gradient of perceptions, specific species seem to be related to the management and use of the area.

### Management practices in caívas

Eleven management practices were found for areas of *caívas* (Table 1). Two family units (7 %) stated that their families managed *caívas* in the past (historical management), however due to the legal restrictions by Environmental Brazilian law they prefer not to continue management in forest areas. One informant stated “I maintain *caívas* out of tradition, it was the way my father had always done it so I do it too”.

**Table 1:** Management practices found in *caívas* according to 28 family units from communities of the Northern Plateau, Santa Catarina, Brazil

Management practice	Description	% family units
Firewood	Pick up firewood from forest floor for personal use. Removing firewood by cutting trees is now illegal but they still depend on firewood so they remove fallen trees or branches from the caiva.	100 % (28)
Cattle	Maintain cattle within caiva to clean herbaceous cover and feed on native pastures. Most families have between 5–20 heads of cattle grazing within caivas. The cattle only graze on native pastures during the summer months, in the winter they also supplemented with oats.	93 % (26)
Trimming	Removal of herbaceous cover with a scythe.	93 % (26)
Pruning/collection of *erva-mate*	Pruning erva-mate with a machete or scissors, or breaking by hand.	93 % (26)
Plant *erva-mate*	Planting erva-mate (*Ilex paraguarienesis*) that grows in the shade within areas of caivas.	79 % (22)
Mowing	Removal of herbaceous cover with tractor or gas powered weed cutter.	79 % (22)
Plant other species	Planting other species, such as *Pinus ellioti, Eucalyptus* sp., *Maytenus* spp (espinheira santa), *Persea major* (pau-de-andrade) and *Pincramnia parvifolia* (pau-amargo) within areas of caivas or forest.	54 % (15)
Pruning	Pruning other species, mainly (*Curitiba prismatica*), with machete or scissors to reduce size.	39 % (11)
Favoring erva-mate	Favoring erva-mate within caiva or forest area in order to increase its growth, making sure the species survives over others.	36 % (10)
Favoring other species	Favoring other species within caiva or forest area, such as *Maytenus* spp. and *Araucaria angusfolia*, making sure the species survives over other.	4 % (4)
Chop firewood	Cut down trees for firewood for personal use.	4 % (1)

The most common management practices within *caívas* were the gathering of firewood from fallen trees and branches, cattle grazing on native pastures in the forest understory, the trimming of the herbaceous layer, and the extraction of erva-mate. All family units remove the herbaceous layer yearly to facilitate the extraction of erva-mate leaves and to create easier access to native pastures for cattle. The cattle also help maintain this area clean and clear of herbaceous cover.

The extraction of erva-mate is done by 93 % (26) family units every 2–5 years. The gathering of erva-mate used to be a communal activity, however, erva-mate industries are now hired for this process. The leaves are “sold on the tree” by 39 % (11) of family units, where the family sells the leaves of the trees that the erva-mate business removes. Of the 93 % of family units who extract erva-mate, 53 % gather their own erva-mate and sell the leaves to the erva-mate industries.

Other species are pruned in order to reduce tree size, generally because the species is creating too much shade for the erva-mate or reducing space for the erva-mate to grow. The cattle’s function is not only to graze on native pastures but also to help maintain the area clear of ferns and other herbaceous species. Only one species was mentioned specifically in relation to pruning, which was the cerninho (*Curitiba prismatica*). This species is a fast growing shrub, which 82 % of family units called a “pest”. These family units went on to say that the species has taken over their *caívas* and that they must remove the species yearly. Two participants said they favor cerninho because it is a species with a hard core to be used as wood for building fences.

Ten family units (36 %) favor erva-mate, that is, they favor this species over others within the *caíva*, making sure of its survival. Four family units (14 %) also said they favor other species. The other species cited were *Maytenus* spp (espinheira-santa) and *Araucaria angustifolia* (araucaria). Firewood is essential, all families have traditional wood stoves, and during the winter temperatures may drop to zero degrees, so the wood stove is essential for household heating. All families said their firewood is from the *caíva*. As firewood they use fallen branches and trees, only one family unit said they also cut down trees for firewood.

### Plant resources from caívas

**Fig. 3:**
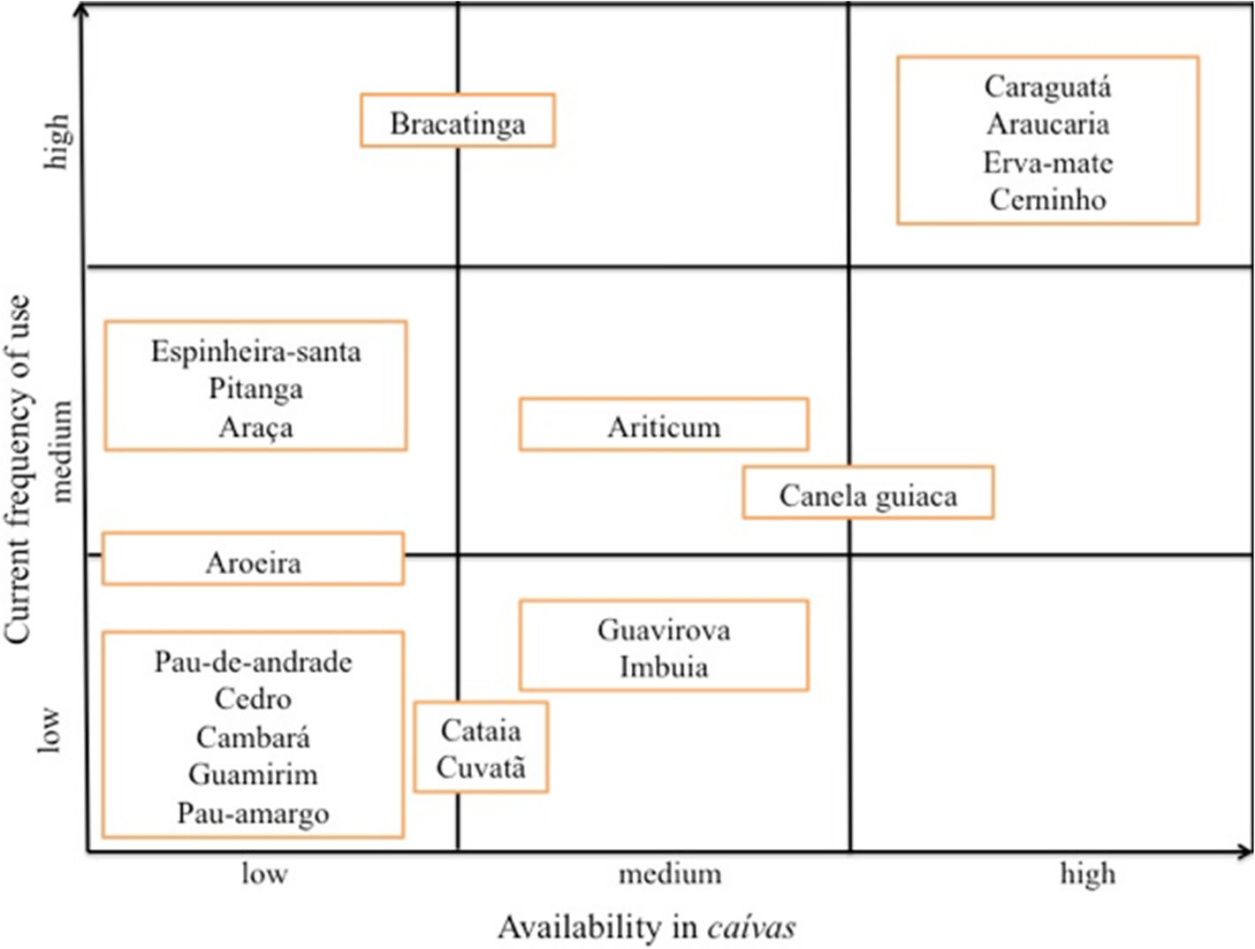
Nine-cell analysis demonstrating the distribution of twenty native species recognized as priority within *caívas*. Analysis was conducted according to availability and current use frequency of 28 family units from the northern plateau of Santa Catarina, Brazil

Twenty native species previously recognized by the local farms as priorities within *caívas* are displayed in the nine-cell analysis (Fig. 3). The analysis shows the distribution of the species according to how frequently it is used and its availability within *caívas*. The species that are said to be highly abundant are also used with a higher frequency, and the species that are not readily available are used with a low frequency. However, some species, such as, espinheira-santa (Maytenus spp.), bracatinga (*Mimosa scabrella*), pitanga (*Eugenia uniflora*), and araça (*Psidium cattleianum*) are used with a medium-high frequency but have a low availability. Thirteen out of twenty species are found to have low use frequency and low availability.

Only four species had high frequency of use and high availability, which were cerninho (*Curitiba prismatica*), araucaria (*Araucaria angustifolia*), erva-mate (*Ilex paraguariensis*) and caraguatá (*Bromelia antiacantha*) (Fig. 3).

**Fig. 4:**
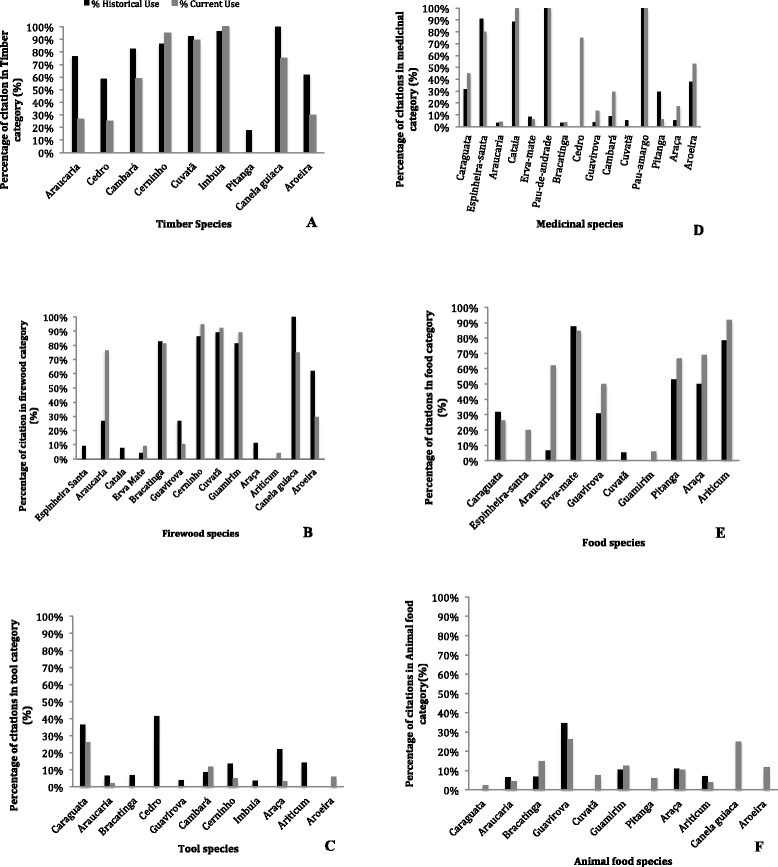
**a**-**f**. Percentage of current and historical use citation for twenty native species considered priority within *caívas*. Analysis conducted from six communities of the northern plateau of Santa Catarina, Brazil. Citations in the following categories: **a** Timber; **b** Firewood; **c** Tool; **d** Medicinal; **e** Food; **f** Animal food

For many of the species the category of use changed temporally, specially in the past 30 years for the different use categories (Fig. 4a-f). There has been a general decrease in current use for timber species (Fig. 4a). For example, araucaria was used almost 80 % solely for timber historically, but the most cited use currently for this species is as food (the araucaria’s seed *pinhão*). In general citations for species used as timber resource decreased from historical use to current use, with the exception of cerninho, cuvatã and imbuia. For firewood (Fig. 4b) the same pattern can be found. Species that were used for firewood historically have changed. There are some exceptions, such as the araucaria that has a larger current use rather than historical use, since its fallen branches are used for firewood. Thus, farmers do not have cut down trees as done in the past. The other exceptions are the bracatinga, a species historically only used for firewood, guamirim, cuvatã and cerninho, which have replaced other species that were used historically for firewood. The use of species cited in the tool category has currently decreased. Most of the species used to make tools are cited currently as no longer used or have decreased in use over time (Fig. 4c). For medicinal plants, the current use and historical use has remained relatively the same, with the exception of cedro (*Cedrela fissilis* Vell.) (Fig. 4d). Cedro was cited in the past as primarily (95 %) timber species but currently is only cited as a medicinal species (80 %). Food species have remained the same in terms of current use and historical use (Fig. 4e), with the exception of the araucaria, which has currently increased in citation, compared historically. Animal food has also generally remained the same between current and historical use citations (Fig. 4f). Some species have appeared currently as being used for animal food that did not appear historically for this purpose. Four out of the eleven species cited in this use category are from the Myrtaceae family (guamirim, pitanga, araça, and guavirova), which was stated by family units to provide fruit for livestock along with the native pastures within *caívas*.

### Spatial and temporal changes in caívas

All family units stated that there have been many changes to the management practices in *caívas* and to the *caíva* itself within the last thirty years. There are three inter-related categories of changes, economic, cultural and legislative, that can be analyzed regarding the changes faced by the local farmers.

The most stated change, due to environmental legislation in general, cited by 57 % of family units, was related to the ability to collect firewood from the *caíva*. More specifically, they recounted that they were no longer allowed to use bracatinga (*Mimosa scabrella*), which used to be their primary source of firewood. Ten family units said they also sold bracatinga wood before it was prohibited by law. All participants said that the worst thing the environmental law disallowed was the use of fire in forest areas and thus the bracatinga is becoming less common (see also Fig. 3).

The second most cited change, which is economic, stated by 54 % of family units, was the decreasing value of erva-mate. All said that the erva-mate is not worth as much, economically and culturally, as it was in the past. In the past erva-mate extraction was a collective community activity, stated by 35.7 % of family units. All 53.5 % of family units stated that in the past erva-mate cultivation and extraction was the primary source of income, along with raising cattle in the *caíva*.

Another economic change mentioned by 32 % of family units is the use of timber resources. Most families stated that their houses are all made with wood from their *caívas*, generally *Araucaria angustifolia* and *Ocotea* spp. Formerly participants were able to sell the araucaria to supplement their income. Furthermore, the family units stated this was the biggest change for them, because the araucaria has a high economic value as a timber resource. Almost 18 % of family units stated that they do not conserve the araucaria anymore since its use is prohibited by legislation and the araucaria consumes space for other resources. Instead the araucaria seedlings found within *caívas* are removed, since the species is found on the endangered species list and cannot be cut without authorization after a specific diameter. Thus, many landowners decide to remove the araucaria as a seedling in order not to have problems with legislation in the future. The araucaria seed, *pinhão*, is only seen as a resource to be used within the household and not to supplement income. In relation to changes in species all participants said that the cerninho (*Curitiba prismatica*) is a species that grows abundantly within *caívas*.

Some of the changes stated were that *caívas* do not exist anymore, now forest areas once considered *caívas* must be legally conserved by Brazilian environmental legislation. Furthermore, that *caívas* were of high value historically, both economically and culturally, but do not have the same value currently. One informant only maintains the *caíva* out of tradition, and 14 % used to take greater care of their *caívas* when able to use its resources. One informant stated that, “before the *caíva* was the future and profit, our children’s inheritance, now it’s just capital we cannot use”. Lastly, 14 % said that the *caívas* used to be the source of income for the family but now it has no value.

The third most cited change is cultural and had to do with the division of land, cited by (43 %) of family units, mostly from the community of Campininha. These family units stated that formerly the whole community was one large *caíva* and that there were no property lines or divisions with fences. One informant said, “it was all one land without fences”, another said, “it was a shared area where livestock were all raised together, and no one knew whose pig was whose”. All participants mentioned livestock being raised free within the *caívas* and that they fed on native fruits. In relation to livestock, family units mentioned how cattle and pigs remained within *caívas* year round feeding on native pastures and fruits, and now since the land was divided they had to plant winter crops to feed the animals. One family unit said they only conserve their *caívas* because of their livestock.

When asked what the best use was for the *caíva* currently, the participant stated the following: plant other species like pine and eucalyptus (exotic species), plant bracatinga and guavirova (*Campomanesia* sp.) both good for firewood, plant more native fruit trees, plant espinheira-santa (*Maytenus* spp) and pitanga (*Eugenia uniflora*), exploit timber resources (*Ocotea* spp), improve native pasture areas for cattle, exploit *pinhão* to make flour, increase livestock, increase and conserve araucaria, conserve to exploit timber resources, manage native tree species, there is no more good use, reduce area of *caívas* for cattle crops. The most cited “best use” by 50 % of family units was to decrease amount of cerninho and increase amount of erva-mate within the *caíva*. Another best use cited by 14 % was to change *caívas* into cultivation areas. Lastly, cited by 11 %, to take care of the *caíva* because of the erva-mate and the araucaria.

## Discussion

*Caíva* has been used throughout the northern plateau of Santa Catarina, perceived both through management practices, as well as through the plant resources present, with species from the Araucaria Forest. *Caívas* are ecotopes in a cultural landscape of the Araucaria Forests, modified and transformed through management practices and extraction of natural resources. Reis and collaborators [25] also consider landscapes with the species *A. angustifolia* and *A. araucana* as cultural landscapes, where the presence of these trees reflect use patterns that do not merely serve practical purposes, and instead these landscapes play a key role in forming the identity of the communities who use them. The *caívas* found within Araucaria Forests are not seen merely as forest fragments with management practices, but rather a place that is maintained out of tradition where plant resources can be used or planted and traditional management practices are exerted. The focus of landscape ethnoecology is on how people perceive their landscapes, through local knowledge and management practices [2]. This approach is concerned with not only ecological factors but also cultural and anthropogenic factors of ecotopes. In this study the perspective of landscape ethnoecology allows the ecotope *caíva* to be seen by most families as a complex association between native vegetation of the Araucaria Forest combined with management practices, which includes the extraction of non-timber forest products. Maintaining *caívas* is a cultural tradition for the people of the northern plateau and these ecotopes are considered historical places. *Caívas* make their link with the land, historically from timber products, and currently from non-timber forest products like erva-mate. *Caívas* may be considered continuing landscapes when considering the UNESCO [4] definition of cultural landscapes, since they are still evolving with the local communities today, and the use of this landscape continues to play a role in both the history, future and identity of the communities.

Landscape transformation can be seen through the management practices of removing the herbaceous layer. The cattle within caívas play an important role in this transformation, cleaning the herbaceous layer year round, feeding on many herbaceous plants and keeping the understory free of plants that may interfere with the growth of the erva-mate. Cattle generally do not consume erva-mate plants, however they consume many fruits, which are provided by many native trees such as, araça (*Psidium cattleianum* Sabine), pitanga (*Eugenia uniflora* L.), guamirim (*Myrcia* sp.), cerninho (*Curitiba prismatica* (D.Legrand) Salywon & Landrum), and guavirova (*Campomanesia* sp.) Cattle grazing may even promote tree regeneration [38], principally in Araucaria Forests where bamboo is sometimes densely found, specifically in areas without cattle [39], and bamboo may impede tree regeneration [38, 40, 41]. Most fruits consumed by cattle belong to the Myrtaceae family. The family Myrtaceae is generally found to contribute the most to the floristic patterns of the Araucaria Forest landscape [39, 42, 43]. In some subtypes of the Araucaria Forest, the tree strata is primarily composed by Lauraceae family, which occupies much of the middle canopy, and the Myrtaceae and Aquifoliaceae families that occupy the lower canopy layer [19]. In a phytosociological study of Araucaria Forest ecotopes, Mello [39] found that Aquifoliaceae, Lauraceae and Myrtaceae primarily composed the understory of the *caíva* ecotope.

Two species favored by management practices are the erva-mate and araucaria, which provide the people of the northern plateau with a source of income from non-timber forest products, and are culture keystone species (CKS) for the region [44]. In this study these were also two of the four species that were considered to have a high use frequency and availability. The management of erva-mate and the araucaria, more than any other species have transformed the Araucaria Forest landscape, and are the most dominant species the *caíva* landscapes [39]. These two species have been highly favored within this landscape since their products were and for some people still are the primary source of income. In landscape ethnoecology and historical ecology the latter can been seen as a feedback loop, where the landscape has an effect on peoples’ behaviors and peoples’ behaviors has an effect on the landscape [2, 10, 45]. This is truly evident with the use of the erva-mate and the araucaria. People began making a living off of this species and in turn began to favor this species within the forest area, therefore generating its abundance and cultural symbol.

The removal of firewood from the forest floor, either of fallen trees or branches is very important for those living in the northern plateau. This is not only a management practice within *caívas* but was mentioned as a significant change, most people stating that they could not use the species bracatinga (*Mimosa scabrella*) anymore, which previously was their primary source of firewood. The bracatinga is a fast-growing leguminosae tree species and dominates the early stages of succession; this species is also used for charcoal production in the northern plateau [46]. The bracatinga is a species of conservation interest for local communities in the northern plateau; some of them speak adamantly about the decline of this species, stating, “The bracatinga cannot be found anymore like before, it is going to disappear”. As seen in the nine-cell analysis, the bracatinga has a high use frequency but its availability is considered to be low.

Steenbock [47] worked directly on the use and social aspects of the bracatinga. In his research, he found that the bracatinga has a high economic and social value, and the species is characterized as a human artifact, and a product of gradual landscape domestication. He also found that the use of fire is common in the management of bracatinga, and to form dense areas of bracatinga called *bracatingais*. However, fire is not necessarily needed in order to grow bracatinga, removal of trees and soil disturbance is sufficient to promote bracatinga germination without fire, however, the removal of trees is costly and there is a greater number of germination when the are is burned [47].

Fire is a management tool that has transformed landscapes in many places [6, 48–51] through slash and burn agricultural practices [5], as well as the management of specific species [5, 52]. Fire is one of the few human actions that can alter landscape so intensely, having the capability of drastically changing the structure and composition of forests [52]. In the Atlantic Forest, as well as the Araucaria Forests, anthropogenic fires have played a major role in its mosaic, as it is used to clear areas for crops, pastures [27, 53], and in some areas of the northern plateau, for the bracatinga [47].

Many participants mentioned changes in law since this caused a profound change in how they view and manage *caívas* and plant resources. Various legislative changes modified how people culturally manage erva-mate, plant resources utilized, as well as the end of the *caíva* for some family units. Conservation of the Atlantic Forest has become very important, and the Atlantic Forest Law was defined in 2006 for the conservation of this biome. The Atlantic Forest Law was designed to conserve and regulate the use and management of remaining forest fragments [54]. The law states that the Araucaria Forests are part of the Atlantic Forest Biome and therefore the native remnants of all vegetation types within the Atlantic Forest in primary and secondary regeneration stages (initial, middle, and advanced) will have its use and conservation regulated by law [54].

The Brazilian Forestry Code has also affected and changed the family units perceptions and relationship with *caívas*. Legal reserves and permanent protected areas (APP’s) are established under the Brazilian Forestry Code that has, as its objective, the sustainable development and use of native vegetation. The Forestry Code states that all rural properties must maintain an area of native vegetation; property owners must maintain 20 % of native vegetation [55]. Many of the family units have already changed their lands to legal reserves thereby discontinuing the management practices that once were tradition within *caívas*.

However, *caívas*, through the perception of the local communities, is not what Marques and collaborators [30] considered, an ecosystem with naturalized or native pastures. Through the perception of those who own *caívas*, it is not merely considered a forest fragment where some management is exerted, it is a place that would not exist without management, thus, not all Araucaria Forest fragments are considered *caívas. Caívas* are places of tradition passed along and conserved through generations, where native vegetation is conserved because people use and rely on these resources for their daily lives, not only as a direct source of income, but indirectly through cattle grazing. Once people believed *caívas* were the future and now most people want to change *caívas* into cultivation land, since the law has discontinued many the use of forest resources and many management practices. The *caívas*, and it’s native vegetation, only exist today because people have used and managed these areas, and continue to use the resources provided by the Araucaria Forest, therefore maintaining a cultural tradition.

*Caívas* are another example of a cultural landscape, from many that can be found around the world [4], and is continuing to change with time. The *caíva* landscape combines the work of nature with that of humans. *Caívas*, like many cultural landscapes, demonstrate humans’ intricate relationship with nature, conserving these spaces through use. *Caívas* reflect the use and management techniques of the people of the Northern Plateau of Santa Catarina and further exemplify the necessity of local ecological knowledge in conservation of cultural landscapes.

## Conclusions

*Caívas* are diverse and can be considered an ecotope in a cultural landscape mosaic. The use and management of species has changed over the years due to diverse historical factors in Brazil for the communities of the northern plateau of Santa Catarina. For example, araucaria changed from a timber resource to a food source and the cultural connotations of erva-mate collection have also changed due to new worker laws, as well the fact that bracatingas cannot be used as firewood as had been previously done in the recent past.

Species that were historically used as timber resources are no longer used. Species that were not historically timber resources became new timber resources because of their abundance, such as, cerninho. Species that were food resources continue to currently be seen as a food source, for humans and livestock. Many feel the loss of the ability to use the resources found within their own properties therefore not promoting them to conserve the area, as stated by many family units “when we were able to use the resources we took better care of our *caívas*”. Erva-mate continues to be one of the primary reasons why the *caíva* still exists, as well as tradition and the use of the *pinhão*. However, most of the people of the northern plateau do not see *caívas* as a viable option due to the lack of ability to use resources and would prefer to turn these remnants into cultivation areas. Once the *caíva* provided an economic resource, with araucaria and erva-mate, as well as other resources, and without the use of these species the caíva becomes a low economic source for the family. Landscape ethnoecology studies are important not only to value the local ecological knowledge, but also to understand the perception of communities in regards to ecotopes to better inform management practices that conserve forest areas. The participants perception is that *caívas* were once an ecotope laden with resources, and therefore were conserved, and now without these resources the *caívas* have no value to its owners and should be converted to crop or cultivation. There must be conservation of areas through regulated use that is easy to access by local communities. It would also be valuable to further investigate the relationship of the local communities with Brazilian environmental legislation, since it was mentioned throughout interviews but was not studied in depth. Other studies should also be done to further link the different views of management practices and plant resource use from the Araucaria Forest with socioeconomic and cultural information. The local communities of the northern plateau have conserved these areas, and sometimes have even increased plant diversity through generations, and hope to pass this to generations to come. Their local knowledge of the forest and its uses is extensive and should be considered when aligning public policies to conservation practices.

## Abbreviations

FLONA: National forest
UFSC: Federal University of Santa Catarina
ICMBIO: Chico Mendes Institute for Biodiversity

## Footnotes

1 Conservabio is a project conducted in during the years 2008–2010. The project Conservabio was done through partnerships between the Núcleo de Pesquisas em Floresta Tropicais (NPFT) at Federal University of Santa Catarina, Embrapa (Brazilian Company for Agricultural Research), EPAGRI (Company for Agricultural Research and Rural Extension in Santa Catarina) and ICMBio (Chico Mendes Institute for Biodiversity Conservation). For more information on the project Conservabio please see references [56] and [57].
